# ‘Spirituality’ in Health Studies: Competing Spiritualities and the Elevated Status of Mindfulness

**DOI:** 10.1007/s10943-019-00773-2

**Published:** 2019-02-26

**Authors:** Maria Nita

**Affiliations:** Department of Theology and Religion, University of Birmingham, Birmingham, B15 2TT UK

**Keywords:** Spirituality, Science, Religion, Mindfulness, Health, Secularisation

## Abstract

The article investigates discourses of ‘spirituality’ in the field of Health Studies, among scholarly voices and the voices of the practitioners and patients these studies reflect. It examines current trends in contemporary spirituality as well as links with debates involving science, religion and secularisation. The article argues that, in the public domain, ‘spirituality’ is beginning to denote a collective practice rather than an individual search for meaning. Furthermore, the article identifies some common understandings of spirituality in the context of Health Studies and health environments, such as it being a tool that can facilitate closeness and emotional exchanges. Finally, it proposes that the success and, as I will show, elevated status of ‘mindfulness’ in this field points to ‘competing spiritualities’, despite shared understandings.

## Introduction

The link between spirituality, health and healing is well established since contemporary spirituality is reported by scholars to be marking its territory and making its most important impact via alternative healing practices (Bowman 2000; Corrywright 2009). However, in this article I am mostly interested in scholarly discourses preoccupied with so-called allopathic health practitioners and patients’ voices in mainstream health environments. What is apparent from the reviewed literature is that when scholars in the field of Health Studies talk about spirituality they refer to particular practices and sometimes assume a common understanding of what the term ‘spirituality’ denotes. Based on the articles reviewed for the purpose of the present investigation, spiritual practices are often understood to be such diverse activities and actions as: prayers, rituals, practicing mindfulness, taking part in religious observances, expressing feelings and displaying emotional literacy, talking and connecting with others in meaningful ways—to name some of the most important ones.

I will show here that some practices are more successful or prominent than others in this field, such as mindfulness meditation. Some scholars suggest that the recent recognition of and interest into such spiritual practices as mindfulness, is congruent with ‘a contemplative turn’ towards subjectivity in our contemporary world (Ergas 2014) and even proof of a ‘postsecular age’ (Taylor 2007). I will suggest in turn that the success of mindfulness meditation could be seen as proof of competing processes within the field of spiritual practices in health environments despite the all-inclusive tone. One important polarisation is that between spirituality and religion, because spirituality is often perceived as universal, and therefore free of cultural bias and dogmatism, as opposed to institutionalised religion. Spirituality is increasingly recognised as being capable of making a positive contribution to both mental and physical health (Park 2007; Seybold 2007). Thus, in his *Explorations in Neuroscience, Psychology and Religion*, Seybold (2007) draws on rigorous scientific research and shows that religion and spirituality can serve as important coping mechanisms in this context by offering social support and inclusion in social networks (Koenig 2002), by sustaining lifestyle changes (Powell et al. 2003) and by promoting positive emotions (Seeman et al. 2003), such as forgiveness, and an overall optimistic outlook.

As per James ([1902] 1982), those of us who are ‘morbidly minded’, by which James means a psychological constitution which is incompatible with a religious perspective that can provide meaning and purpose, might not entirely benefit from all of the above—yet this should not invalidate what Seybold recognises here as the material proof for the importance of religion and spirituality in restoring and maintaining health—William James’ so-called fruit of religion. My article will endeavour to examine the contemporary connotative field spirituality has aggregated in the context of Health Studies—but not before looking more broadly at some contemporary understandings of spirituality in specialist literature from other fields of study concerned with religion and culture.

## ‘Spirituality’: Contemporary Understandings and Trends

In the last couple of decades, contemporary spirituality has been the subject of some debate in the field of Religious Studies, particularly regarding the distinction between religion and contemporary spirituality in relation to secularisation, the privatisation of religion and changes to social life (Heelas 1998; Heelas and Woodhead 2005; Pearson 2002; Sutcliffe and Bowman 2000; Taylor 2010). Although scholars from other disciplines, as it is the case with the field of Health Studies, seem to be writing about ‘spirituality’ in a more matter-of-factly fashion, as soon as they attempt to define ‘spirituality’ or propose ‘spiritual guidelines’ they slowly come to encounter the same problems the fields of Anthropology, Sociology and Religious Studies have long confronted, they stumble across such questions as ‘what is spirituality actually?’ and (if we cannot be sure of what it is) ‘what does it do’? An unspoken divide is represented by whether scholars, tacitly or explicitly, believe that ‘spirituality’ does in fact denote something real, that can be explored—or not.

Some criticisms involve the very methodological assumption scholars make when they study ‘religion’ or ‘contemporary spirituality’. Hence, writing as a critical insider, Timothy Fitzgerald (2010) argues that there is no non-theological or non-ideological basis for Religious Studies as a discipline since by affirming that its domain of study is ‘religion’, it implicitly recognises it (‘religion’) as a substantive category that can be defined or investigated. However, the phenomena we refer to as ‘religious’, Fitzgerald argues, is a Western construct rooted in our Judeo-Christian roots which we export and impose on some non-Western cultures and attempt to frame their political or economic structures within our ideological dichotomies, such as ‘religion’ and ‘secularity’ (Fitzgerald 2010, p. 10). This debate can be of course extended to ‘spirituality’: what do theologians, scholars and various circles and networks actually mean by it?

It is not important I would argue to explain what spirituality is—as long as it is possible to investigate the manifestations and connotations associated with ‘spirituality’. We can investigate spirituality or spiritualities as semantic or semiotic fields, as symbolic and material discourses, which can enable us to recognise what ‘spirituality’ may represent for a particular group of people, in a particular place and at a particular time.

Some scholars claim that spirituality can be understood as a multivalent global phenomenon. Thus, in her *Search for Spirituality: Our Global Quest for Meaning and Fulfilment*, Ursula King (2009) talks about a great variety of spiritualities that share some ground ethical and mystical ideals, whether this is spirituality within a religious tradition, personal spirituality, ecological spirituality, interfaith spirituality, feminist spirituality and so on. Albeit a hopeful understanding of spirituality as a global construct that could emerge as a uniting force in a divided and consumption oriented world, this concept appears to be theological, drawing heavily on Christian understandings.

A more analytical attempt at distinguishing traditional spirituality from contemporary spirituality comes from Stuart Rose’s article (2001) which asks ‘is spirituality a word that everyone uses but nobody knows what anyone means by it?’ Here, Rose addresses this question by analysing a survey questionnaire from leaders coming from what he understands as traditional (the big six religious traditions: Judaism, Christianity, Islam, Buddhism, Hinduism and Sikhism) and non-traditional (e.g. Shamanism) contexts. Rose finds some common dimensions, such as ‘connection with the Divine’ and ‘awareness’. However, the very attempt to understand so-called non-traditional, contemporary spirituality, by investigating the viewpoints of ‘leaders’ is anachronistic—since networks of ‘spiritual seekers’ do not follow traditional leadership models (York 1995).

The debate around secularisation continues to use such terms as ‘science’, ‘spirituality’ and ‘religion’ as two-dimensional or monolithic concepts. According to the secularisation thesis (Martin 1978), science was going to be a deciding factor in the process of abandoning obsolete beliefs that had been surpassed by scientific thought. In response to this thesis, the evolutionary psychiatrist Charlton (2006) argues that science, religion and contemporary spirituality—which he calls ‘new age spirituality’—are ultimately complementary, and their successful coexistence in the USA proves this. Charlton argues that the trend towards the relativisation of truth in spirituality (‘truth is what works for me’) and the preoccupation with the social domain in religion, rather than scientific truths, has led to ‘a specialisation in these areas of human experience’, thus occupying different domains and having no need to mix. These are, according to this author, winning strategies that can insure the survival of religion and spirituality in an era increasingly dominated by science. Moreover, Charlton claims that by performing different functions in the modern world, science, religion and spirituality have a relationship of mutual dependence and ‘a bright future’. This view is not really promising in terms of a fertile dialogue since it indicates a relationship of hegemony and marginality, whereby ‘religion’ and ‘spirituality’ can continue to be tolerated, as long as they occupy the private rather than the public sphere.

Despite the fact that some scholars accept this model of separate spheres where religion, spirituality and secularity all occupy distinct fields in the private and public arenas, some theorists argue that if we are experiencing a blurring of the religious versus secular boundaries, it is because we have entered a postsecular age (Taylor 2007) which is happening through the media, the arts and in society at large. Thus, the philosopher Charles Taylor argued that there had been an important turn in modern culture defined by the importance of subjective experience (Taylor 1991). In this view, contemporary society has entered a contemplative turn, marked by a shift in the way people live their lives, from living life by following external guidance to a life that is guided by inner experience. This is ‘an age in which the demarcations between religion, spirituality, and secularity and their relations with education and science become blurred’ (Ergas 2014, p. 59). What is more—mindfulness is credited for spearheading this profound change—through its success and the authority it has earned in public life (ibid.). I will turn to this after investigating a particular noteworthy trend: a move from an individual to a collective dimension in contemporary spirituality.

### Spirituality from an Individual to a Collective Dimension

In my previous research, I recognised that an important postmillennial trend has been represented by a shift from an individual to a collective spirituality (Nita 2016, pp.163–186). Previously spirituality has been perceived as highly individualistic and as such it was seen as proof of secularisation. Thus, for many scholars writing at the turn of the millennium the decline of religion and the growth of spirituality were presumed to be related processes (Heelas 1998; Smart 1998, pp. 572–592). Apart from the declining numbers in religious practitioners, an important British trend was recognised by the British sociologist of religion Grace Davie as ‘believing without belonging’ to any religious institution and in turn taken to signify proof of private religion or alternative spirituality (Davie 1994, pp. 93–116). In their *Spiritual Revolution: Why Religion is Giving Way to Spirituality*, Heelas and Woodhead (2005) distinguished religion from spirituality by juxtaposing the beliefs and practices of a Christian congregation to that of a network of spiritual seekers. They contended that whilst religion was concerned with objective roles, duties and obligations, spirituality was concurrent with a ‘subjective turn in the modern culture’ and is mainly predicated on inner, subjective life. From this stand point

the spiritual revolution can be said to take place when ‘holistic’ activities having to do with subjective-life spirituality attract more people than do ‘congregational’ activities having to do with life as religion. (Heelas and Woodhead 2005, p. 7)

Spirituality was therefore often understood as a loose, floating compound of beliefs and practices divorced from religious traditions, and predicated on the self (Heelas 1996, p. 21; Melton 1992). Moreover, spirituality was often seen as alternative or fringe—a notion that has robustly been contested by scholars, both by challenging the mainstream/alternative boundary (Pearson 2002, pp. 1–12) but also by looking more holistically at the religious scene of the twenty-first century in a historic context. In their editorial introduction to *Beyond New Age: Exploring Alternative Spirituality*, Sutcliffe and Bowman (2000) suggest that contemporary spirituality can be understood as vernacular, folk, religion:

[a]cademic studies of religion […] have tended to concentrate on ‘official religion’, concerned primarily with theology, philosophy and group ritual. ‘Popular’ and ‘folk’ views and practices outside this fairly narrow focus have been treated as quaint, mistaken, superstitious or deviant depending on the context. (Sutcliffe and Bowman 2000, p. 6)

An important trend in contemporary spirituality is its countercultural ethos, well reflected in the ‘spiritual but not religious’ tag. Thus, the growth of contemporary spirituality may be driven by the countercultural shift against institutionalised and colonial religion, and to this end, Suzanne Owen shows that:

‘Native Americans say they are employing the term „spirituality” as a reaction to missionary religions, associated with colonialism, but [also as the] move toward „spirituality” and away from institutional forms of religion is also part of a wider trend in Western society.’ (Owen 2008, p. 5)

In my own research with climate activists (particularly during protest festivals) I found that green spirituality had developed a communitarian dimension reflected in artistic and performative activities (Nita 2016). Similarly other festivals, such as Burning Man, seen by insiders as a festival about creativity, spirituality and community, does not accord well with an understanding of ‘spirituality’ as individualistic, subjective and predicated on the self, which might indicate that ‘spirituality’ is once again on the move, coming to describe new collective values and relationships. Green spirituality and festival spirituality are examples of a collective or shared spirituality that is beginning to articulate a common praxis, sometimes at the expense of religion as some scholars have suggested (Taylor 2010). Creative developments inside the green movement, with communal ritual practices, shared artistic expressions, lantern processions and many other similar practices, may suggest that in the public domain contemporary spirituality is no longer understood to be predicated on the self, but it is evolving towards a more collective, communitarian expression.

I turn now to investigating how spirituality is understood in the field of Health Studies, by reviewing and examining scholarly literature in this area.

## Talking About ‘Spirituality’ in Health Studies and the Success of Mindfulness

Few studies among those reviewed for the purposes of the present article, namely a large section of articles from the field of Health Studies, published in the last two decades and dealing with spirituality, deviated from the simple conclusion that there is an urgent need to integrate knowledge and understanding about spirituality into health care. In his *Medicine, Religion, and Health: Where Science and Spirituality Meet*, Koenig (2008) makes a case for scientists to consider the important contribution spirituality can make to health. Koening emphasises the correlation between spirituality, mental and physical health and advocates for the development of clinical applications, to combat stress, anxiety and depression, promote well-being and positive emotions. In support of this proposal, Koenig discusses a large number of studies that, after controlling for other factors such as lifestyle and religiosity, clearly accounted for improved health, increase immune function and better recovery outcomes.

Whilst in the 1990s we can still find studies that absolutely negate the usefulness of spirituality for mental and physical health (Walters 1992), in recent studies even the most neutral voices recommend some spiritual engagement and spiritual literacy on the part of the physician or psychiatrist (Lake 2012). Thus, most studies in the twenty-first century, concerned with a variety of health problems, recognise both religion and spirituality as having a role in maintaining health and recovery (Seybold 2007). For example, according to some studies religious patients complied better with follow-up treatment and had less anxiety and fewer health worries (Casar Harris et al. 1995). Another study entitled ‘Do patients want physicians to inquire about their spiritual or religious beliefs if they become gravely ill?’ (Ehman et al. 1999) found that a majority of patients for whom spirituality was important wanted their physicians to address their spiritual beliefs. Therefore, in this literature spirituality and religion are simply conflated, and there is rarely a consistent distinction between the two. Even if the author makes a distinction, they hardly follow up this distinction in the studies they end up quoting. Furthermore, patients appear to use these terms interchangeably.

As already noted, Seybold (2007) examined numerous scientific studies looking at the mechanisms at work in the success of religion and spirituality. He showed that social networks, healthier lifestyles and increased optimism, were some important factors. For most authors, it seemed less important to establish whether this was a placebo effect, and some studies acknowledge the placebo aspect whilst still affirming the benefits of this possible effect. For instance, Herbert Benson, a cardiologist at Harvard School of Medicine, refers to the placebo effect in this instance as ‘remembered wellness’ (Benson 1996).

The interest scientists have shown healing practices and spirituality is sometimes related to testing these with the view of investigating their claims (Bomar 2013; Brown 2014; Hewson et al. 2014). Despite some mixed results, many studies emphasised benefits in complementary healing practices, even if these were not evidenced based. For example, when assessing data obtained from participants, studies have found that prayer (Brown 2014) or healing ceremonies (Hewson et al. 2014) can have positive outcomes on alleviating symptoms and improving quality of life.

Among the various spiritual practices addressed by such studies, mindfulness distinguishes itself as a particular area of focus, with many scholars and practitioners recommending mindfulness training programs to achieve stress reduction (Byron et al. 2015; Kabat-Zinn 2003; Pipe et al. 2009). Schools, universities and even the army (Myers 2015) often support mindfulness projects and training programmes, and it is somewhat surprising that mindfulness has achieved this level of credibility in mainstream circles, considering how ‘spiritually sanitised’ public spaces have become. To illustrate this point, please see the following announcement from the School of Nursing E-Newsletter, at the University of Pittsburgh:

On 16 January and 13 February this year [2014], the University of Pittsburgh Medical Center (UPMC) in the USA conducted two mindfulness meditation retreats under the heading “The Practice of Mindfulness: A Retreat to Promote Self-Care for the Professional Nurse, Educator, and Leader, to Understand the Role of Mindfulness Meditation to Enhance the Delivery of Nursing Care. (Holtz 2014)

The association with leadership, which is there in scholarly literature as well (Pipe et al. 2009), seems to be a means of promoting mindfulness as a successful mainstream practice and counteract any possible damaging reminders that mindfulness may also be considered a spiritual practice, and thus carry countercultural, fringe and alternative undertones.

New scientific research in this area confidently shows that mindfulness training is correlated with stress reduction, brain plasticity and gene expression (Creswell et al. 2012; Giuliani et al. 2011; Kaliman et al. 2014; Larouche et al. 2015). One study conducted in the USA suggests that mindfulness has an effect on gene expression and can therefore reduce morbidity in older adults (Creswell et al. 2012). Similarly, a parallel study from researchers at the University of Wisconsin and the University of Barcelona (Kaliman et al. 2014) found that mindfulness has applications in regulating inflammatory pathways as well as regulating gene expression, a fact that was considered a promising breakthrough for future research.^1^

The prominence of mindfulness in scientific investigations and its success in allopathic medicine could be due to the specific properties mindfulness meditation actually has and may illustrate the real applications of so-called spiritual practices, more broadly. It is, however, possible to argue that the popularity of this practice and the recent high-quality research into mindfulness has led to high-quality results. The success of mindfulness can be attributed to a Western, Cartesian bias represented by a preference towards ‘a mind spirituality’ over a ‘body spirituality’. Alternatively the success of mindfulness can be discussed from the perspective of the ‘easternisation of the west’ (Campbell 2010), and ensuing rejection of traditional Christian practices. In either case, the success of mindfulness in this field could indicate that rather than speaking about spirituality, it may be more accurate to talk about ‘competing spiritualities’, and future research could consider spiritual practices in the context of power relationships: if leaders practice mindfulness, who practices Reiki or aura healing for example?

## Spirituality and the Health Practitioner

Despite the scholarly support for spirituality evidenced above, when it comes to speaking about spirituality, practitioners seem less inclined to address or inquire into their patients’ spiritual needs or offer any practical help. Speaking from a double scholarly practitioner perspective Gedge and Querney (2014) called spirituality ‘the silent dimension’, because it is often left out from important conversations with patients and carers, particularly in the context of addiction treatment.^2^ According to these authors, although practitioners are prepared to address difficult subject areas such as sexual abuse, trauma and addiction, talking about spirituality involves an ethical midfield that is carefully avoided by clinicians. The authors call for the formulation of ethical guidelines that would inform clinicians on how spirituality can be integrated into professional practice. Therefore, in this case spirituality is identified as a resource and as a way of navigating or talking about painful subjects.

The importance of being able to communicate about religion and spirituality between patients and care givers is powerfully illustrated in the following example from Puchalski (2001):

When I was a resident I saw a 28 year-old woman whose husband had just left her. She found out that her husband had AIDS, and she asked to be tested. When I met with her to tell her that the test result came back positive, I tried to explain that her illness was diagnosed early and that there had been recent advances in the treatment of HIV that were allowing people to live longer with their illness. She kept referring to God and about why God was doing this to her. I recognized that we weren’t connecting, so I asked her about her comments. She proceeded to tell me about being raped as a teenager and having an abortion. In her belief system, that was wrong. I remember her exact words: “I have been waiting for the punishment, and this is it. (Puchalski 2001, p. 355)

Puchalski concludes that integrating spirituality into health care is part of delivering a compassionate care and fully listening to patients (Puchalski 2001, p. 357).

What is perhaps unsurprising is that spirituality is already far better integrated into health care in non-Western countries and research shows that this has clear positive effects. This may be because religion and spirituality are not ‘silent dimensions’ in countries where they are not overtly set in opposition with secularity and therefore pushed into the private sphere. Hence, in a study about how medical doctors use spirituality in their practices in Puerto Rico, Koss-Chioino and Espinosa (2013) show that doctors were open to discussing spiritual matters and even open to be guided by their own spirituality when diagnosing or recommending investigative procedures. One doctor, for example, reported feeling the patient’s illness as a ‘pain’ in her soul, an interesting description that could be discussed in terms of empathy, intuition, understanding and compassion, qualities that seem to be part of the connotative field of what is understood as spirituality in this field.

There is also a growing interest and scholarly persuasion for the medical field to include spiritual insights into its approach to healing and (again) to recognise the emotional needs of the patients. A wealth of evidence from empirical research published in medical journals shows that ‘spirituality’ has a positive effect on patients, carers and even medical practitioners, particularly when it comes to integrating spirituality in mainstream/allopathic approaches (Dunn and Horgas 2004; Sirati Nir et al. 2013; Strawbridge et al. 1997; Wachholtz et al. 2007). This is often seen as repairing the more mechanical relationship between medical professional and patient, as well as recognising the importance of having a more empathetic and compassionate approach that can yield real results in recovery and the maintenance of health (Offenbaecher et al. 2013). Therefore, we encounter in this literature a discourse on spirituality as a tool for understanding and discussing emotional needs—being empathetic and demonstrating compassion.

Spirituality is reported as an important factor in coping with disease and maintaining quality of life. A study investigating factors influencing end-of-life decisions in patients with gynaecologic cancer conclusively shows that women depended on their religious convictions and experiences to cope with the disease (Roberts et al. 1997). A large percentage of those surveyed (93% of 108 women) cited having spiritual beliefs and 75% of these patients said they were more spiritual after diagnosis (Roberts et al. 1997, p. 72). In this case, spirituality appears to be understood by patients as something one gains through experience, sadly, as a result of the difficult experience of coping with disease.

The literature concerned with caring for the spiritual needs of gravely ill patients suggests that there is a discrepancy between the high level of scholarly interest in this field and the low level of acknowledgement in the medical field for the need of a spiritual care. Back in the early 1990s, Ross (1994) noted that although nurses were charged with spiritual care for their patients and despite evidence that spirituality could improve recovery and promote well-being, the lack of guidelines in the medical field was stunting progress. As I have shown this remains the complaint from many practitioners and scholars today. Some authors propose that guidelines would encourage nurses to engage in spiritual practices with patients if this were appropriate and in harmony with their own authentic spiritual beliefs (Winslow and Winslow 2007). In a qualitative study in nursing in Holland, Van Leeuwen et al. (2006) show that despite the lack of attention to this type of care, spiritual care is a reality of the relationship between nurses and patients and that a common religious language helps nurses and patients communicate about such important issues as life, death, health, family and pain. In Van Leeuwen’s study, older and more experienced nurses were recognised by patients as more able to communicate about spiritual needs, a fact that might suggest that nurses learn or gain ‘spiritual skills’ as they interact with patients. Van Leeuwen’s work suggests that it is of crucial importance to be able to communicate across religious boundaries with patients who are suffering acutely and/or experiencing loss or trauma. Spirituality is thus understood as an important skill, gained through experience, a lingua franca which enables health practitioners to communicate around important matters, such as health, family, pain and death.

As I conclude the present article, my local hospital has just opened a new ‘spirituality care centre’. Their website announces this as follows:

Our brand new Spiritual Care Centre – a space for prayer and reflection – has now opened. It replaces our former Chapel and offers 24-hour access for people of all faiths or none, providing room for anyone to come and sit, to talk, reflect, pray or simply gather their thoughts. Ablution and prayer spaces are available for those who need them, as well a quiet and private area for confidential and sensitive conversations. There’s also a peaceful courtyard garden with planting, seating and sculpture. [The] Lead Chaplain […] says: “It is a real privilege and exciting to be in this new space. We’re looking forward to working in our modern, purpose-built centre, providing religious and spiritual care to people with diverse needs from all backgrounds, cultures, all faiths and none.^3^

**Fig. 1:**
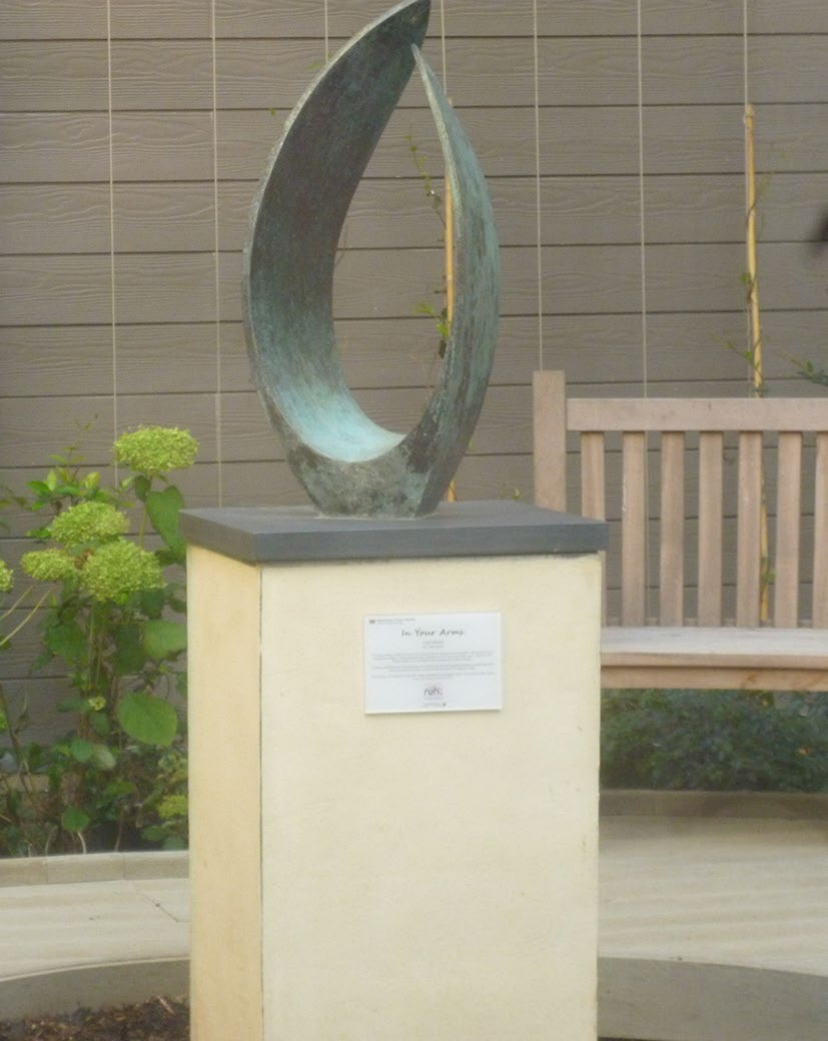
‘In Your Arms’, The Spiritual Care Centre, The Royal United Hospital, Bath, UK. Copyright Maria Nita

The language in this announcement is as inclusive as it can be, clearly trying to cater for those of different faiths and none—and thus replacing the more Christian sounding Chapel with a Spiritual Care Centre. The space is described as ‘brand new’, ‘modern’, ‘exciting’, ‘purpose-built’—making this change more evident. I visited the centre and found a lovely large room with comfortable chairs, a central tall table shaped like an altar, a shielded area covered in colourful prayer rugs, various decorative objects, abstract art that reminded one of the stained glass windows of a church. Two large doors led to a lovely garden area and the central sculpture, entitled ‘In Your Arms’ (Fig. 1), clearly stays away from all key religious symbols: it is not reminiscent of a star, a cross, a crescent, or a wheel. The arms of the sculpture come together in what looks like an embrace and almost touch. It suggests care and closeness to me.

## Conclusion

My analytical review suggests that despite scholarly debates and controversies around science, secularisation, religion and spirituality, and about the meaning of spirituality, in the field of Health Studies and furthermore, in mainstream, allopathic health environments, scholars, practitioners and patients have, or at least presume to have, shared ideas and a common understanding of what spirituality is. My review of key scholarly works published in the last two decades showed that ‘spirituality’ had a largely positive connotative field, being associated with recovery, compassion, empathy and emotional knowledge. I found that the terms spirituality and religion were often used interchangeably, yet the term spirituality is clearly preferred by scholars for its inclusive connotation. There is a clear indication that spirituality in this context is often considered, by patients and practitioners alike, a valuable and hard-earned tool or resource. Moreover, spirituality is often understood as an enhanced ability to communicate with patients, which is gained through experience.

My examination showed that scholars claimed that the contemplative or subjective turn in modern culture is changing our approach to health and vice versa. I contended here that the success and elevated status mindfulness enjoys in Health Studies raises some important questions about what this is actually doing to or doing for other spiritual practices. Research and future studies could investigate if and how mindfulness practices may be competing with other forms of spirituality, as some of the literature investigated here suggests. A preference for mindfulness might suggest that this practice is more compatible with institutional secularisation. Future studies could explore what other specific forms of spirituality are making a mark in the areas of health and healing and how may these developments be impacting on science. Ethnographic studies in these areas could also investigate the material culture of spirituality in health environments, looking at how prayer rooms or spirituality care centres, pictures, icons, sculptures and other ‘spiritual’ objects, movements or sounds may be used and understood in allopathic health environments.

Finally, I showed that whilst religion and science may have a history of competition and conflict, and whilst some scientists may still be concerned with testing and debunking the value of spirituality in health and healing practices, the recent trend has been that of investigating the benefits of spirituality, sometimes regardless or in the absence of scientific evidence. Thus, it seems that whilst for many people science, religion and spirituality may appear to be at odds with each other, this is no longer important—and perhaps never was—when people are talking meaningfully about pain, life and death. The implications for Health Studies are however complex and my article wished to highlight an urgent need for qualitative research in this area, looking at how ‘spirituality’ may be framed, understood and constructed.

## Footnotes

1 This study speaks of ‘the therapeutic potential of mindfulness-based interventions’ and claims to ‘set the foundation for future studies to further assess meditation strategies for the treatment of chronic inflammatory conditions’ (Kaliman et al. 2014: 96).

2 Gedge and Querney do not necessarily advocate a spiritual intervention in addiction treatment, but they show the limitations of current clinical practice when patients’ recovery depends upon the satisfaction of their own spiritual and religious needs (Gedge and Querney 2014: 48).

3 A press release from the Royal United Hospital in Bath, UK [online]. http://www.mnrjournal.co.uk/article.cfm?id=107496&headline=New%20Spiritual%20Care%20Centre%20opens%20in%20Bath%C2%A7ionIs=news&searchyear=2018.
